# Quality of life in bipolar disorder: A review of the literature

**DOI:** 10.1186/1477-7525-3-72

**Published:** 2005-11-15

**Authors:** Erin E Michalak, Lakshmi N Yatham, Raymond W Lam

**Affiliations:** 1 Department of Psychiatry, University of British Columbia, Vancouver, Canada

**Keywords:** bipolar disorder, quality of life, literature review

## Abstract

A sizable body of research has now examined the complex relationship between quality of life (QoL) and depressive disorder. Uptake of QoL research in relation to bipolar disorder (BD) has been comparatively slow, although increasing numbers of QoL studies are now being conducted in bipolar populations. We aimed to perform a review of studies addressing the assessment of generic and health-related QoL in patients with bipolar disorder.

A literature search was conducted in a comprehensive selection of databases including MEDLINE up to November 2004. Key words included: bipolar disorder or manic-depression, mania, bipolar depression, bipolar spectrum and variants AND quality of life, health-related QoL, functional status, well-being and variants. Articles were included if they were published in English and reported on an assessment of generic or health-related QoL in patients with BD. Articles were not included if they had assessed fewer than 10 patients with BD, were only published in abstract form or only assessed single dimensions of functioning.

The literature search initially yielded 790 articles or abstracts. Of these, 762 did not meet our inclusion criteria, leaving a final total of 28 articles. These were sub-divided into four categories (assessment of QoL in patients with BD at different stages of the disorder, comparisons of QoL in Patients with BD with that of other patient populations, QoL instrument evaluation in patients with BD and treatment studies using QoL instruments to assess outcome in Patients with BD) and described in detail.

The review indicated that there is growing interest in QoL research in bipolar populations. Although the scientific quality of the research identified was variable, increasing numbers of studies of good design are being conducted. The majority of the studies we identified indicated that QoL is markedly impaired in patients with BD, even when they are considered to be clinically euthymic. We identified several important avenues for future research, including a need for more assessment of QoL in hypo/manic patients, more longitudinal research and the development of a disease-specific measure of QoL for patients with BD.

## Review

Good quality of life (QoL) encompasses more than just good health. At a basic level, it can represent the sum of a person's physical, emotional, social, occupational and spiritual well-being, the study of which is complicated by the fact that there is no consensus as to what constitutes QoL. The World Health Organization has described QoL as "individuals' perception of their position in life in the context of the culture and value systems in which they live and in relation to their goals, expectations, standards and concerns" [1]. This broad, generic conceptualization of QoL can be distinguished from the more specific concept of 'health-related quality of life' (HRQOL), which refers to those aspects of an individual's life that impact directly upon their health [2] and the more economically-derived 'cost-utility' models of QoL. This area of research is further complicated by the understanding that QoL can be highly subjective, potentially fluid and open to distortion, making it challenging to measure reliably and accurately. Yet, there is a growing body of evidence to suggest that QoL is an important indicator of well-being, and one that we should be attempting to capture when assessing the patient health.

The assessment of QoL in medical settings may be of value in several ways. QoL instruments can provide levels of information not always supplied by traditional outcome measures. For example, some instruments such as the Schedule for the Evaluation of Individualized Quality of Life (SEIQoL) [3] and the Patient Generated Index [4] allow patients to prioritize which life domains are most important to them. While the reduction of symptoms may be the primary goal of the clinician, it may be that the patient places more emphasis upon restoring family relationships, or being able to engage in leisure activities. These 'individualized' measures, although sometimes difficult to administer and interpret, put the patient at the centre rather than at the periphery of assessing the effectiveness of treatment interventions. QoL assessments can also help determine patient preference, allow comparisons of well-being between different conditions and detect subtle differences in response to treatment that may be missed by traditional outcome measures.

While a host of studies have now examined QoL in patients with major depressive disorder (MDD) (for example, [5-8] until recently few had specifically focused upon QoL in patients with bipolar disorder (BD). The slow uptake of QoL research in BD may have occurred in part because of the absence of a 'disease-specific' measure of QoL for bipolar populations, or because of reservations about the ability of patients with BD to reliably and accurately complete self-report measures, particularly when in a manic phase.

Two reviews of previous research addressing health-related QoL (HRQOL) in BD have been conducted [9,10]. In the first of these, Namjoshi and colleagues (1999) assessed all relevant English-language articles published prior to 1999, identifying 10 studies for inclusion. The studies proved to be quite heterogeneous, and used a variety of generic and depression-specific instruments to assess different aspects of HRQOL. They also tended to be relatively small (only one study had a sample size in excess of 100 patients), were conducted in depressed or euthymic (rather than hypo/manic) patients, and rarely included descriptions of the psychometric properties of the instruments they utilized. The authors of the review made a number of suggestions for future research, including the development of a disease-targeted measure of QoL for BD, more assessments in acutely manic patients, and more longitudinal research. The second review conducted by Dean and colleagues (2004) examined studies that had assessed HRQOL, work-impairment or healthcare costs and utilization in patients with BD published prior to November 2002. The review applied a very broad definition of HRQOL, including in this category studies that had assessed social or physical functioning in isolation (for example, the Global Assessment of Functioning or GAF scale was included as a measure of HRQOL). Using this broad definition, the review identified 65 HRQOL articles. The authors concluded that deficits in HRQOL in patients with BD are similar to those observed in patients with unipolar depression and equal or lower than levels of HRQOL observed in patients with other chronic medical conditions.

Given the recent upsurge of interest in describing QoL in BD, the present study aimed to provide an updated literature review of studies that have assessed both generic and HRQOL in patients with bipolar disorder.

## Materials and methods

A comprehensive literature search (supplemented by hand searching where appropriate) was conducted in the following databases up to November 2004:

MEDLINE (1966–2004)

EMBASE (the Excerptra Medica database) (1988–2004)

PubMed (1967–2004)

PsychINFO (1967–2004)

CINAHL (Cumulated Index to Nursing and Allied Health Literature) (1982–2004)

American College of Physicians Journal Club (1991–2004)

CDSR (Cochrane Database of Systematic Reviews) (-2004)

CCTR (Cochrane Controlled Trials Register) (-2004)

DARE (Database of Abstracts of Reviews of Effectiveness)

IPA (International Pharmaceutical Abstracts) (1965–2004)

Key words used for the search included: bipolar disorder or manic-depression, mania, bipolar depression, bipolar spectrum and variants AND quality of life, health-related QoL, functional status, well-being and variants. Articles were included if they were published in the English language, and reported on the assessment of generic or HRQOL in patients with BD. Our definition of QoL was not overly-inclusive; we required that studies had used a QoL or HRQOL scale that assessed several domains of functioning. Studies using scales that examined single domains of QoL (for example, those assessing solely social or occupational functioning, or single-item scales such as the GAF) were excluded. We omitted studies that included fewer than 10 patients with BD, but did not reject reports for other scientific limitations (for example, convenience sampling or cross-sectional designs). Studies that were underway but were not completed were excluded, as were conference abstracts, dissertations or reports on QoL in BD that were not published in peer-reviewed journals. We also excluded studies that reported assessments in groups of patients with heterogeneous diagnoses where results for patients with BD were not reported separately, and where individual results this population could not be provided by the authors after personal communication (for example, [11-25].

## Results

**Figure 1:**
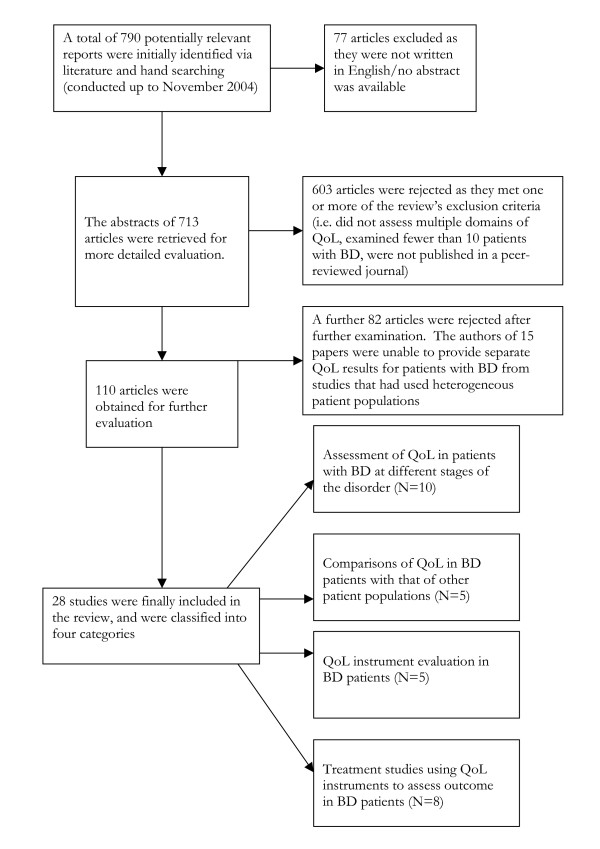
Flowchart of review results.

The results of the literature search are summarized QUOROM-style in Figure 1. The final 28 included articles are summarized in Table 1.

**Table 1:** Summary of studies assessing quality of life in patients with bipolar disorder

**Study**	**Location**	**Population(s)**	**QoL instrument(s)**	**Main findings and limitations**
Arnold et al., (2002)	US	44 BD patients (38 type I, 5 type II, I NOS) 30 back pain patients 2474 general population	SF-36	HRQOL impaired in BD patients compared to non-clinical sample. Chronic back pain patients more impaired in all SF-36 domains except role limitation (emotional) and mental health. Limitation – disparate sample sizes.
Atkinson et al., (1997)	Canada	37 BD patients 69 patients with schizophrenia 35 MDD patients	QoL index	BD and MDD patients subjectively reported lower QoL than patients with schizophrenia, but schizophrenia group had poorer objectively measured QoL. Limitation – relatively small BD and MDD sample sizes.
Bond et al., (2000)	US	149 patients with SMI (21 with BD)	QOLI	Mean overall life satisfaction QOLI scores showed mid-range impairment. Limitation – small sample of patients with BD.
Chand et al., (2004)	India	50 BD patients in remission 20 patients with schizophrenia 20 control subjects	Q-LES-Q, WHO-QOL-BREF	Patients with BD generally reported better QoL than patients with schizophrenia, and equivalent QoL to control group subjects. Limitation – incomplete matching between groups; unusually low Q-LES-Q scores in control group
Cooke et al., (1996)*	Canada	68 euthymic BD patients (55 type I, 13 type II)	SF-20	SF-20 scores comparable to those reported for patients with MDD. BD type II patients reported poorer HRQOL that BD type I. Limitation – shortcomings of SF-20 compared to SF-36.
Dogan et al., (2003)	Turkey	26 outpatients with BD stabilized on lithium	WHO-QOL-BREF	Significant improvement in general health, physical functioning and social functioning 3 months after a psychoeducation intervention. Limitation – small sample size.
Kusznir et al., (2000)	Canada	61 euthymic BD patients (47 type I, 14 type II)	OPQ	One third of sample did not meet criteria for adequate community functioning. Limitation – cross-sectional research design.
Leidy et al., (1998)	US	62 BD patients, type I (34 euthymic, 28 depressed)	SF-36, QLDS, MHI-17 and CFS	Psychometric properties of instruments generally in acceptable ranges. Marked impairment in SF-36 scores apparent and QLDS scores lower than reported elsewhere for patients with unipolar MDD. Limitation – test-retest reliability was measured over an unusually long period.
MacQueen et al., (1997)	Canada	62 euthymic BD patients, type I	SF-20	No significant differences in SF-20 scores between psychotic and non-psychotic patients. Limitation – small sample of patients with psychotic symptoms.
MacQueen et al., (2000)	Canada	64 euthymic BD patients, type I	SF-20	Number of previous depressive episodes a stronger determinant of HRQOL than number of previous manic episodes. Limitation – number of previous episodes determined retrospectively.
Namjoshi et al., (2002)	US	139 BD patients, type I	SF-36	Acute treatment with olanzapine resulted in improved SF-36 physical functioning scores; improvement in vitality, pain, general health and social functioning domains apparent in open-label phase. Limitation – adjunctive use of lithium and fluoxetine during open-label phase.
Namjoshi et al., (2004)	US	224 BD patients, type I	QOLI	Olanzapine cotherapy associated with better outcome in several QOLI domains compared to monotherapy with lithium or valproate. Limitation – only acute QoL outcome data available.
Olusina et al., (2003)	Nigeria	25 outpatients with BD type I or II	WHO-QOL-BREF-TR	Majority of sample report 'fair/average' QoL. Small sample of patients with BD, little clinical information for sample.
Ozer et al., (2002)	Turkey	100 interepisode BD patients	Q-LES-Q	Depression scores on SADS interview significantly predicted lower Q-LES-Q scores. Limitation – cross-sectional nature of research.
Patelis-Siotis et al., (2001)	Canada	49 BD mildly depressed or euthymic patients	SF-36	SF-36 vitality and role (emotional) scores significantly improved after CBT. Limitation – Open study, and SF-36 scores only available for a sub-set of patients.
Perlis et al., (2004)	US	983 patients with BD type I, II or NOS	Q-LES-Q	Younger age of onset of BD predicts Q-LES-Q scores.
Revicki et al., (1997)	US	28 outpatients diagnosed with DSM-III-R BD	SF-36	Onset of BD determined retrospectively. No significant differences in SF-36 domain scores according to mode of administration (in-person vs. telephone). Limitation – small sample size.
Revicki et al., (2003)	US	120 BD type I patients hospitalized for acute mania	Q-LES-Q	No differential effects of treatment with divalproex sodium vs. olanzapine on QoL Limitation – only 43% of randomized patients completed Q-LES-Q
Ritsner et al., (2002)	Israel	17 BD patients (9 manic, 4 depressed, 4 mixed)	Q-LES-Q and LQOLP	Q-LES-Q scores poorest in depressed patients, highest in manic. Limitation – small sample of patients diagnosed with BD.
Robb et al., (1997)*	Canada	68 euthymic BD patients (55 type I, 13 type II)	IIRS	Greater illness intrusiveness associated with higher Ham-D scores, recent depression and BD type II. Limitation – IIRS not validated for use in BD populations.
Robb et al., (1998)*	Canada	69 euthymic BD patients (54 type I, 15 type II)	SF-20	Women possessed significantly lower SF-20 scores in the domains of pain and physical health. Limitation – shortcomings of SF-20 as a HRQOL measure.
Russo et al., (1997)	US	241 BD inpatients (138 depressed, 103 manic)	QOLI	Manic BD patients reported better QoL than BD depressed patients. Limitation – lower response rate in acutely manic group.
Ruggeri et al., (2002)	Italy	22 BD patients	LQOLP	LQOLP mean scores similar to those observed in larger mixed sample of psychiatric patients. Limitation – small sample of bipolar patients.
Salyers et al., (2000)	US	164 BD patients	SF-12	Mental health scores significantly lower in patients with unipolar depression. Limitation – brief nature of SF-12.
Shi et al., (2002)	Europe US, South America South Africa	453 BD patients, type I	SF-36	Olanzapine superior to haloperidol in improving HRQOL during acute and continuation treatment in most SF-36 domains. Limitation – relatively high drop-out rates during acute treatment phase.
Shi et al., (2004)	7 countries	573 BD in/outpatients, type I, most recent episode depressed	SF-36, QLDS	Olanzapine-fluoxetine combination associated with grater improvement in HRQOL. Limitation – high drop-out rate for an 8-week trial (55%).
ten Have et al., (2002)	Netherlands	136 BD patients (93 type I, 43 NOS)	SF-36	BD sample generally showed greater impairment in SF-36 scores than patients with other psychiatric diagnoses. Limitation – accuracy of CIDI diagnosis of BD NOS in question.
Tsevat et al., (2000)	US	53 BD patients	SF-36, TTO and SG	TTO (0.61) and SG (0.70) scores for mental health comparable to those reported for other psychiatric conditions. Limitation – cognitive complexity of TTO and SG tasks.
Vojta et al., (2001)	US	86 BD patients (16 manic/hypomanic, 26 MDD, 14 mixed, 30 euthymic)	SF-12 and EuroQoL	SF-12 mental health scores significantly lower in manic group than in euthymic group. MDD/mixed group SF-12 scores significantly poorer than in manic/euthymic groups. Limitation – small sub-samples, brief nature of the SF-12.
Wells et al., (1999)	US	331 BD patients 944 double depression 3479 MDD 151 dysthymia 987 depressive symptoms	SF-12, TTO and SG	BD group had lower health utility than MDD, dysthymia and depressive symptoms groups. Limitation – cognitive complexity of TTO and SG tasks.
Yatham et al., (2004)	15 countries	920 BD type I patients (currently depressed/experienced episode of depression in previous 60 days)	SF-36	SF-36 scores markedly impaired compared to general population norms and consistently lower than sub-scale scores for patients with unipolar MDD. Limitation – depression severity not controlled for.

* counted as one study for purposes of review

EuroQoL visual analog scale

Illness Intrusiveness Rating Scale

Lancashire Quality of Life Profile

Lehman Quality of Life Interview

Longitudinal Interval Follow-up Evaluation

Mental Health Index 17

MOS Cognitive Function Scale

MOS Short Form 12

MOS Short Form 20

MOS Short Form 36

Occupational Performance Questionnaire

Quality of Life Enjoyment and Satisfaction Questionnaire

Quality of Life in Depression Scale

Quality of Life Index

Quality of Life Interview

Severe Mental Illness

Standard gamble

Time tradeoff

World Health Organization Quality of Life Assessment

### Review of studies

This section will review the 28 studies we identified. For ease of interpretation they are classified into the following four categories, although several studies met criteria for more than one category.

i) Assessment of QoL in patients with BD at different stages of the disorder

ii) Comparisons of QoL in patients with BD with that of other patient populations

iii) QoL instrument evaluation in patients with BD

iv) Treatment studies using QoL instruments to assess outcome in patients with BD

#### i) Assessment of QoL in patients with BD at different stages of the disorder

We identified ten studies of QoL in patients with BD at different stages of the disorder. Four of these were generated by a research group in Canada, and will be dealt with in unison. Following this, six other studies (one comparing QoL in patients with during different phases of the disorder, a recent study assessing QoL in bipolar depression, one performed in a Turkish sample of interepisode patients, one conducted in a sample of patients attending a mental health service in Italy, one in recently discharged patients in Nigeria and a report on patients enrolled in the STEP-BD Program) will be described.

A research group in Toronto, Canada has generated a series of interrelated reports on QoL in BD. Three of the series [26-28] describe various aspects of QoL in a single sample of outpatients (N = ~68) with BD type I (with manic episodes) or II (with hypomanic episodes) who had been clinically euthymic for at least one month (these have been counted as one study for the purposes of this review). Three of the series report on QoL in other patient populations [29-31]. Cooke and colleagues [26] examined levels of HRQOL using the MOS SF-20, [32] a self-report questionnaire designed to assess perceived well-being in six domains (physical, social and role functioning, mental health status, health perceptions and bodily pain). Mean scores on the SF-20 domains in study patients were comparable to those reported for patients with MDD by Wells and colleagues in the large RAND Corporation MOS Study [33]. Analysis of SF-20 scores by type of BD showed that patients with BD type II reported significantly poorer HRQOL than BD type I in the areas of social functioning and mental health. In another paper, Robb and colleagues [27] reported on functioning in the context of the 'Illness Intrusiveness Model' in patients with BD [34,35]. The model addresses the impact a disorder and/or its treatment has upon an individual's activities across 13 life domains: health, diet, active/passive recreation, work/financial status, self expression/improvement, family relations, relations with spouse, sex life, other relationships, religious expression and community involvement. The Illness Intrusiveness Rating Scale (IIRS) is used to yield a 'total illness intrusiveness' (TII) score. Illness intrusiveness occurred in several areas of functioning, with TII being associated with higher Hamilton Depression Rating Scale (Ham-D) scores, patients having experienced a recent episode of depression and having type II BD. Robb and colleagues [28] specifically focused upon gender differences in SF-20 scores, finding that women possessed numerically lower scores in all of the questionnaire's domains except for mental health, with significant differences in the domains of pain and physical health. Interestingly, objective measures of functioning (clinician rated Global Assessment of Functioning or GAF scores) were not significantly different by gender.

MacQueen and colleagues [29] examined SF-20 scores in euthymic BD type I patients (N = 62) with or without psychotic symptoms during an index episode of mania. No significant differences in SF-20 scores were apparent between patients with or without psychosis, although the sample identified with psychosis may have been too small (N = 16) to detect statistically significant differences between sub-groups. Kusznir and colleagues [30] assessed levels of community functioning via the Occupational Performance Questionnaire (OPQ) in a similar population, finding that one-third of patients did not meet criteria for adequate functioning on the 'Community Functioning Scale' component of the questionnaire. Finally, MacQueen and colleagues [31] focused upon the effect of number of manic and depressive episodes on SF-20 and GAF scores in euthymic patients (N = 64), finding that number of past episodes of depression was a stronger determinant of HRQOL than number of previous manic episodes. Good correlation between the subjectively rated SF-20 and objectively rated GAF scores provided some evidence that euthymic patients with BD are capable of providing accurate descriptions of their HRQOL.

A potential advantage of this series of studies is that the majority of them were conducted in euthymic outpatients; interepisode patients are likely to be less prone to the effects of cognitive distortion than are symptomatic patients. However, euthymic patients are not necessarily asymptomatic as many have mild sub-syndromal symptoms, and several studies in this review will demonstrate that even residual depressive symptoms can be strongly associated with impaired QoL. The relationship between QoL and hypo/mania is less well understood. Both mania and hypomania can be associated with substantial depressive symptomatology, either in the form of 'dysphoric mania/hypomania' or when the patient experiences a mixed episode. This understanding led Vojta and colleagues to hypothesize that patients with manic symptoms would report significantly lower QoL than would patients who were euthymic [36]. To test this theory, the authors administered two brief self-report measures (the SF-12 and the EuroQoL visual analog scale) in bipolar patients with mania/hypomania (N = 16), MDD (N = 26), mixed mania/hypomania and depression (N = 14) or who were euthymic (N = 30). In keeping with their hypothesis, patients with mania/hypomania did show significantly lower SF-12 mental health scores than euthymic patients, with depressed or mixed patients showing significantly poorer HRQOL again. Mean EuroQoL scores ran in the same direction, although the difference between euthymic and manic/hypomanic patients was not significant.

In the largest study to date of QoL in bipolar depression, Yatham and colleagues have reported on SF-36 scores in BD type I patients (N = 920) who were either currently depressed, or had experienced a recent episode of depression [37]. SF-36 scores were remarkably low in the role-physical, vitality, social functioning, role-emotional and mental health sub-scales (see Table 2). The authors went on to compare these scores with those derived from seven large (>100 outpatients) studies of HRQOL in unipolar depression that had also administered the SF-36. Sub-scale scores tended to be lower in the bipolar sample than in the unipolar sample, with the exception of the bodily pain sub-scale, where unipolar depressives tended to exhibit higher scores. Mean SF-36 scores were significantly (weakly: range -0.1 to -0.3) negatively correlated with HAM-D scores, providing some evidence for the construct validity of the instrument in this population. Whilst this study is robust in terms of its large sample size and well-described clinical population, it did not control for depression severity or demographic variables in between-group comparisons. Furthermore, diagnosis of bipolar disorder was made by careful clinical interview, whereas unipolar depression was diagnosed via a number of subjective and objective methods.

**Table 2:** Summary of studies using the SF-36 to assess quality of life in patients with bipolar disorder^1^

Study	Patient population	Physical	Social	Role physical	Role emotional	Pain	Mental health	General health	Vitality
Arnold (2000)	44 BD outpatients	78.8 ± 22.4	57.9 ± 27.7	63.1 ± 41.6	38.6 ± 43.1	64.9 ± 25.7	55.3 ± 23.8	61.9 ± 25.4	43.6 ± 24.3
Have (2002)^2^	93 BD type I 43 BD NOS	89.6 91.2	73.6 80.8	77.6 81.7	69.5 80.6	74.1 82.5	62.3 68.7	62.6 68.2	58.0 62.0
Leidy (1998)	34 euthymic 28 depressed	84.4 ± 20.2 72.2 ± 28.3	73.2 ± 18.2 29.3 ± 20.0	86.2 ± 28.0 32.3 ± 38.6	76.2 ± 31.2 8.3 ± 20.3	59.6 ± 29.0 54.7 ± 25.3	69.2 ± 17.9 33.4 ± 16.5	70.9 ± 20.7 58.0 ± 21.2	52.0 ± 16.2 20.4 ± 17.5
Namjoshi (2002)	122 BD type I (manic/mixed) 65 olanzapine 57 placebo	86.8 ± 16.8 84.5 ± 21.9	47.1 ± 28.3 46.0 ± 31.8	70.4 ± 40.2 65.4 ± 40.3	37.4 ± 42.3 36.3 ± 43.3	68.4 ± 26.4 61.7 ± 25.0	59.9 ± 22.6 58.5 ± 19.8	69.0 ± 22.7 65.2 ± 24.3	63.3 ± 24.0 66.6 ± 20.0
Patelis-Siotis (2001)	34 BD CBT completers 8 BD CBT non-completers	80.4 ± 19.3 63.8 ± 30.6	58.1 ± 25.0 46.9 ± 28.1	41.2 ± 39.8 40.6 ± 44.2	17.6 ± 33.1 29.2 ± 41.5	68.5 ± 23.7 63.4 ± 27.0	52.4 ± 18.0 44.0 ± 22.0	66.6 ± 21.7 46.4 ± 29.6	39.4 ± 19.3 28.1 ± 21.4
Revicki (1997)	14 BD patients (in-person) 14 BD patients (by telephone)	78.4 ± 25.2 77.0 ± 29.3	53.6 ± 30.2 57.1 ± 29.9	65.2 ± 38.7 59.8 ± 41.0	40.5 ± 42.9 33.3 ± 40.6	68.0 ± 31.8 69.3 ± 28.2	53.4 ± 22.8 53.9 ± 20.0	59.8 ± 22.8 57.5 ± 22.7	41.4 ± 18.7 41.4 ± 20.5
Tsevat (2000)	53 BD patients	78.7 ± 23.4	58.7 ± 27.9	63.2 ± 40.9	38.9 ± 42.3	65.3 ± 26.0	56.2 ± 23.7	62.1 ± 24.3	45.4 ± 24.4
Shi (2002)	453 BD type I 234 olanzapine 219 haloperidol	85.2 ± 23.2 90.5 ± 15.7	61.1 ± 31.8 61.2 ± 29.1	66.1 ± 39.6 72.8 ± 36.3	53.3 ± 43.1 50.1 ± 43.7	79.8 ± 26.2 81.2 ± 26.1	71.0 ± 20.4 72.8 ± 16.5	73.6 ± 21.8 75.1 ± 19.2	75.8 ± 19.1 80.0 ± 14.9
Shi (2004)	573 BD type I (currently depressed) 250 olanzapine 58 olanzapine/fluoxetine combination 265 placebo	65.8 ± 27.6 68.8 ± 25.0 66.6 ± 26.2	29.1 ± 20.9 30.6 ± 20.8 32.5 ± 21.4	47.8 ± 44.0 44.8 ± 41.8 46.4 ± 42.3	12.9 ± 25.4 9.8 ± 23.4 14.6 ± 28.7	60.6 ± 27.1 60.8 ± 25.6 57.8 ± 26.1	30.0 ± 16.1 31.0 ± 17.3 31.3 ± 15.7	51.1 ± 22.3 52.3 ± 20.7 48.6 ± 22.6	25.5 ± 17.5 25.3 ± 19.0 25.6 ± 17.6
Yatham (2004)	920 BD type I (currently depressed/ depressive episode in previous 60 days)	70.2 ± 26.2	29.9 ± 22.8	36.7 ± 40.9	11.4 ± 23.5	62.2 ± 27.1	31.0 ± 17.3	47.5 ± 23.3	22.4 ± 17.7

1 Data presented as mean ± SD

2 SDs not available

A study by Ozer and colleagues [38] assessed 100 interepisode patients with BD in Turkey with the aim of examining the impact of 'history of illness' and 'present symptomatology' factors upon a variety of outcome measures including the Schedule for Affective Disorder and Schizophrenia (SADS) and Quality of Life Enjoyment and Satisfaction Questionnaire (Q-LES-Q) [39]. The Q-LES-Q is a 93-item self-report measure of the degree of enjoyment and satisfaction in various areas of daily living. The questionnaire was developed and validated for use in depressed outpatients and has eight summary scales that reflect major areas of functioning: physical health, mood, leisure time activities, social relationships, general activities, work, household duties and school/coursework. Mean Q-LES-Q scores can be derived from the eight summary scales and range from 0–100, where higher scores indicate better QoL. Using multivariate analysis, Ozer and colleagues found that none of the historical variables (including age at first episode, number of previous depressive/manic episodes, duration of illness, number of hospitalizations, age at first hospitalization, or number of symptoms during first episode) were predictive of mean Q-LES-Q scores. Of the current symptoms assessed, only the depression subscale of the SADS interview significantly predicted lower Q-LES-Q scores, accounting for only 13% of the observed variance. When the patient population was subdivided into three groups (low, moderate and high) according to severity of SADS depression scores, mean Q-LES-Q scores were 39%, 38% and 35%, respectively. In comparison, mean Q-LES-Q scores have been reported to be 42% in hospitalized psychiatric inpatients [40], 42% in outpatients with MDD [41], 44% in patients with seasonal affective disorder (SAD) [41], 53% in patients with chronic MDD [42], and 83% in the general population (Rapaport, personal communication).

Ruggeri and colleagues [43] investigated the relationship between QoL and a variety of clinical and demographic variables in a community-based sample of patients (N = 268) with mixed psychiatric diagnoses, 22 of whom were bipolar. QoL was assessed via the Lancashire Quality of Life Profile (LQOLP), which assesses perceived well-being and functioning in 9 major life domains on a 7-point Likert scale (where higher scores indicate better QoL). We extracted LQOLP results for the bipolar sample from data provided by the authors, finding that mean satisfaction scores for the 9 domains were 4.4 ± 1.0, a score similar to that reported for the entire patient sample. In another study of recently discharged Nigerian outpatients (N = 25) with BD type I or II, World Health Organization Quality of Life Assessment (WHO-QOL-BREF-TR) scores were reported to be 'good' in 5 (20%) of patients, 'fair/average' in 14 (56%) and 'poor' in 6 (24%) of patients (data by WHOQOL-Bref domain also provided by authors during personal communication) [44].

Finally, Perlis and colleagues have recently provided an analysis of 'early onset' in 983 patients (BD type I, II or NOS) enrolled in the Systematic Treatment Enhancement Program for Bipolar Disorder (STEP-BD) [45] in which QoL was assessed. The multicentre STEP-BD program, a large prospective, naturalistic study than combines several randomized-controlled trials, has selected to use the Q-LES-Q to assess QoL and the GAF and 'Range of Impaired Functioning Tool' (LIFE-RIFT) to measure functional status. Perlis and colleagues provide the first report on QoL from the project, having looked specifically at the effect of age of onset (grouped into 'very early age, <13 years', 'early age, 13–18 years' and 'adult, > 18 years') of mood symptoms in BD upon outcome. Younger age of onset was found to be a significant predictor of Q-LES-Q scores at study entry (where treatment and clinical status would have varied widely between patients), but not of functioning as measured by the GAF or LIFE-RIFT. These results represent early data from a study that has the potential to address several important questions surrounding QoL in BD.

#### ii) Comparisons of QoL in patients with BD with that of other patient populations

We identified five studies comparing QoL between patients with BD and patients with other conditions. Two of these used the SF-36, one used the 'Quality of Life Index', the Q-LES-Q and the WHO-QOL-BREF and one applied a 'health utilities' model.

The SF-36 [46] is currently the most widely used measure of HRQOL [47]. The self-report questionnaire contains eight sub-scales assessing physical functioning, social functioning, role limitations (physical), role limitations (emotional), pain, mental health, general health and vitality. These yield an overall domain score on a 0–100 scale, where 0 represents worst possible health and 100 best possible health. Arnold and colleagues [48] compared SF-36 scores between patients with BD (N = 44) and chronic back pain (N = 30) with norms previously reported for a general population sample (N = 2,474) [49]. The results of the study indicated that HRQOL was compromised in all SF-36 domains except physical functioning in patients with BD compared with the general population sample (see Table 2). The BD group fared better than the back pain group in the physical, role limitation (physical), pain and social domains, although no significant differences were observed in terms of role limitation (emotional) or mental health domains. While the study provides a useful initial comparison of HRQOL between BD and other conditions, its findings should be interpreted with some caution owing to the disparate sample sizes involved. It also utilized previously published norms for the SF-36 that had been derived by different data collection methods.

The Netherlands Mental Health Survey and Incidence Study (NEMESIS) has examined the epidemiology of psychiatric disorders in a large general population sample [50]. Using the Composite International Diagnostic Interview (CIDI), 136 adults were identified with DSM-III-R lifetime BD (93 with BD type I and 43 with BD NOS) and administered the SF-36. Participants with BD showed significantly more impairment in most of the questionnaire's domains compared with NEMESIS subjects diagnosed with other psychiatric disorders (SF-36 scores for the BD group are presented in Table 2). For example, in the domain of mental health, participants with BD type I experienced significantly lower mean scores (62.3) than people with other mood (75.2), anxiety (74.0), substance use (80.2) or no psychiatric disorders (85.8). BD type I subjects also experienced significantly lower SF-36 scores than patients with BD NOS in the domains of mental health, role limitations (emotional), social functioning and pain. However, there remains some controversy about the accuracy with which the CIDI detects BD NOS, limiting somewhat the inferences that can be made on the basis of these sub-group results. A later analysis of a sub-set (N = 40) of the original NEMESIS sample administered the EuroQol: 5 Dimensions (EQ-5D) scale, which can be used to provide health utility values [51]. Mean utility values (see below) for the sample were reported to be 0.82 ± 0.20, comparable to those observed in the general population of the Netherlands.

Atkinson and colleagues [52] used a different measure, the 'Quality of Life Index' [53], to assess QoL in patients with BD (N = 37), MDD (N = 35) or schizophrenia (N = 69). The authors found that subjectively reported QoL was lower in patients with BD and MDD than in those with schizophrenia. Interestingly, this trend was reversed for objectively assessed QoL, which included measures such as medical history, health risk behaviors, educational and financial levels and social functioning. These findings led the authors to speculate about the validity of subjective measures of QoL, particularly in people with affective disorders. These results were not replicated in Indian by Chand and colleagues, who compared the QoL of patients with BD (in remission and stabilized on lithium prophylaxis, N = 50) with patients with schizophrenia (N = 20) and healthy controls (N = 20) [54]. Using the Q-LES-Q and the WHOQOL-BREF, the authors found that the bipolar group reported significantly better QoL than the schizophrenia group in all domains of the Q-LES-Q, and in general well-being, physical health and psychological health on the WHO scale. Surprisingly, the authors also observed that perceived QoL was equivalent between patients with BD and healthy controls, with the exception of the Q-LES-Q leisure domain, where the patient group actually reported better functioning. Having said this, mean Q-LES-Q scores for this particular control group were unusually low (approximately 47%, where general population norms for the United States are around 83%, Rapaport, personal communication).

Although a growing number of studies have now evaluated the 'health utilities' and 'health preferences' of patients with physical conditions, relatively few have examined these values in patients with mental illnesses, including BD. The concept of health utility refers to an individual's preferences for different health states under conditions of uncertainty. Health preferences are values that reflect an individual's level of subjective satisfaction, distress or desirability associated with various health conditions. Health utility and preferences are frequently assessed by the 'time tradeoff' (TTO) and 'standard gamble' (SG) approaches [55]. TTO refers to the years of life a person is willing to exchange for perfect health. For example, patients might be asked to imagine that a treatment exists that would allow them to live in perfect physical and mental health, but reduces their life expectancy. They might then be asked to indicate how much time they would give up for a treatment that would permit them to live in perfect health, if they had ten years to live. SG refers to the required chance for successful outcome to accept a treatment that could result in either immediate death or perfect health. For example, patients might be asked to imagine that they had ten years to live in their current state of health, and that a treatment existed that could either give them perfect health, or kill them immediately. Patients might then be asked to indicate what chance of success the treatment would have to have before they would accept it. Health utility and preference values are frequently expressed as a score of 0 to 1, with higher values representing better health.

We identified one study comparing health utility in patients with BD with other patient populations. Wells and colleagues (1999) [56] assessed functioning and utility in patients with depression or chronic medical conditions within seven managed care organizations in the United States. HRQOL was assessed via the global mental and physical scales of the SF-12 and utility was measured via TTO and SG. Patients with depression were categorized as those with BD (N = 331), 12-month MDD (N = 3479), 12-month double depression (N = 944), 12-month dysthymia (N = 151) or brief subthreshold depressive symptoms (N = 987). In terms of HRQOL, the bipolar group showed levels of impairment second only to patients with double depression. Utility was also lower in the bipolar group compared with patients with MDD, dysthymia or brief depressive symptoms, although not double depression. In terms of health utility, bipolar patients were willing to give up on average 17% of their life expectancy in return for perfect health, and would accept on average an 11% risk of death in exchange for perfect health. In comparison, patients with MDD were willing to give up 11% of their life expectancy, and accept a 6% risk of death.

#### iii) QoL instrument evaluation in patients with BD

We identified five studies that had evaluated different QoL instruments in BD populations and one study that examined the effects of mode of questionnaire administration. The first of these examined the application of the aforementioned health utility approach. The second assessed the psychometric properties of the Lehman Qualify of Life Interview (QOLI) in a heterogeneous sample of psychiatric inpatients. The third evaluated four QoL scales in a smaller sample of Patients with BD, while the fourth assessed the MOS SF-12 in a large population of patients with severe mental illness. The fifth study evaluated the properties of the Q-LES-Q and the LQOLP in a sample of Israeli patients with severe mental disorders. The final study we identified examined telephone versus in-person health status assessment in outpatients with BD.

Tsevat and colleagues (2000) [57] examined functional status and health utility in 53 outpatients with BD recruited from one site of the multicenter Stanley Foundation Bipolar Network study. The authors aimed to assess how patients with BD rated their current overall health versus their current mental health, and to determine the extent to which health utility correlated with disease state. TTO scores for current overall health were 0.71, but were significantly higher than scores for current mental health, which averaged 0.61. In other words, patients with BD were willing to give up on average 39% of their life expectancy in return for perfect mental health. These values are similar to TTO values obtained in the Beaver Dam Health Outcomes Study in patients with depression (0.70) or anxiety (0.77). SG scores were not significantly different for overall health (0.77) and mental health (0.70). SF-36 scores for the study are presented in Table 2. Certain SF-36 domains (general health, vitality and role-emotional) were significantly correlated with mental health TTO and SG scores, but levels of mania were not correlated with utilities for either overall health or mental health. The authors concluded that health utilities may be related to certain health status attributes and to level of depression, but may not be related to level of mania in patients with BD. One advantage of the health utility/preference approach to QoL assessment is that it allows the calculation of quality-adjusted life years (QALYs). QALYs are a commonly used outcome measure in cost-effectiveness studies, but our literature search did not find any studies that had calculated QALYs for BD populations.

Russo and colleagues [58] performed a rigorous psychometric evaluation of the QOLI [59] in a large sample (N = 981) of acutely ill hospitalized psychiatric inpatients. Of these, 138 were diagnosed according to DSM-III-R criteria with bipolar depression, 103 with acute mania and the remainder with unipolar depression, schizophrenia, or 'other' diagnoses. The QOLI contains 44 items and 7 satisfaction scales, a global satisfaction item and 14 functional items, with all satisfaction scores ranging from 1 (terrible) to 7 (delighted). Patients were administered the instrument using a structured interview procedure within 48 hours of admission and discharge. While the QOLI was successfully completed by 90% of patients overall, rates did vary according to patient diagnoses with non-completion rates being lowest in patients with bipolar depression (12%) and highest in manic patients (31%). Reasons given for non-completion of the measure varied, the most common being 'inadequate staff time' (39%), 'patient too psychotic, demented, or confused' (13%), or 'too agitated or sleepy' (12%). The QOLI showed good psychometric properties overall, although there was some concern about an apparent lack of construct consistency (low correlations between satisfaction and functional measures) in patients with mania. Analysis of QOLI sub-scales showed that, broadly speaking, manic patients reported the highest levels of satisfaction and function, with bipolar and unipolar depressed patients reporting the lowest levels.

Leidy and colleagues [60] examined the psychometric properties of four QoL measures in 62 BD type I patients (34 euthymic, 28 depressed). Patients completed the SF-36, the Quality of Life in Depression Scale (QLDS), the Mental Health Index 17 (MHI-17) and the MOS Cognitive Function Scale (CFS). The study provided further evidence that both euthymic and depressed patients with BD are capable of providing subjective reports of their HRQOL. Baseline SF-36 scores were markedly impaired in the depressed sub-group, with the vitality, social and role limitation (emotional) domains all falling below the 25^th ^percentile (see Table 2). QoL as measured by the QLDS was poorer than has been reported elsewhere for patients with unipolar depression. Cronbach's alpha scores for the QLDS, MHI-17, CFS and four of the eight SF-36 sub-scales (physical functioning, role physical, vitality and metal health) all fell above the generally accepted level of 0.80. Test-retest reliability for the scales were modest (intraclass correlations ranged between 0.18 on the SF-36 role emotional scale and 0.80 for physical functioning), although the reliability of the scales was assessed over an unusually long time period (8 weeks). Scores on the QLDS, MHI-17 and CFS were significantly correlated with patients' Ham-D scores, as were several of the SF-36 sub-scales, thus confirming the construct validity of the scales in patients with BD. Finally, the MHI-17, CFS, QLDS and SF-36 vitality, role emotional and mental health sub-scales were shown to be responsive to change in depression status over time; the QLDS has recently been successfully used as an outcome measure in a large pharmaceutical treatment trial (Shi et al., 2004, see section IV).

Salyers and colleagues [61] conducted a psychometric evaluation of another MOS instrument, the SF-12 [62], in a sample of 946 patients with severe mental illness, 164 of whom were diagnosed with BD. Mean (± SD) SF-12 physical functioning and mental functioning scores for the bipolar group were 46.1 ± 11.5 and 39.6 ± 12.7 respectively, although mental health functioning scores were significantly lower (31.8 ± 13.4) in patients with unipolar MDD. The instrument showed acceptable levels of reliability and validity in the entire sample, although it is worth noting that is was administered by trained interviewers, rather than as a self-report measure.

Ritsner and colleagues [63,64] have compared responses on the Q-LES-Q and the LQOLP in a sample of 175 non-clinical controls and 199 Israeli patients with severe mental illness (SMI), 17 of whom were diagnosed with BD. In personal communication with the authors, we were informed that mean Q-LES-Q scores for the manic, depressed and mixed sub-groups of Patients with BD in the study were 40%, 25% and 33% respectively. Both instruments showed generally acceptable levels of internal consistency, test-retest reliability and criterion validity (in the entire patient population) but notably low levels of convergent validity between the instruments' domains, particularly in the control group. Finally, Revicki and colleagues (1997) [65] examined the effects of administering the SF-36 either in person or by telephone in 28 patients with BD (see Table 2). SF-36 domain scores were not significantly affected by mode of administration.

#### iv) Studies using QoL instruments to assess outcome in patients with BD

We identified eight studies that had used a QoL measure to assess outcome in BD populations: five clinical trials that examined pharmacological interventions for the disorder and three studies that assessed non-pharmacological interventions.

Namjoshi and colleagues from a Lilly research group have conducted a series of studies examining the impact of treatment with olanzapine upon QoL [66-70]. In the first, Namjoshi et al., (2002) evaluated the impact of acute (3-week) treatment with olanzapine or placebo and long-term (49-week open label) treatment of BD type I (manic/mixed). Baseline SF-36 scores for the olanzapine and placebo group are shown in Table 2. During acute-phase treatment, a significant improvement was observed in the physical functioning domain of the SF-36 in the olanzapine group. During the open label treatment period, however, the SF-36 bodily pain, vitality, general health and social functioning domains showed significant improvements over time. This may indicate that olanzapine has a relatively rapid effect in terms of improving physical functioning in patients with acute mania, but that treatment may be required for longer periods for functioning to improve in other QoL domains.

Shi and colleagues have also compared the treatment effects of olanzapine and haloperidol in patients with acute mania (N = 453) [67,68] weeks of acute-phase treatment, significantly greater improvement in five of the SF-36 domains (general health, physical functioning, role limitations – physical, social functioning and vitality) was apparent in the olanzapine group. Superiority of olanzapine over haloperidol persisted over the study's 6-week continuation phase, with concomitant improvements in work and household functioning. Baseline SF-36 scores for the olanzapine and haloperidol groups are shown in Table 2. A further study examined the effects of adding olanzapine to lithium or valproate in patients with BD (N = 224) [69]. Olanzapine cotherapy was associated with better outcome in several QOLI domains compared to monotherapy with lithium or valproate alone. The SF-36 and QLDS have been used in a study comparing the benefits of olanzapine alone versus an olanzapine-fluoxetine combination or placebo [70]. Compared with placebo, patients who received olanzapine showed greater improvement at 8 weeks in SF-36 mental health summary scores, and in mental health, role-emotional and social functioning domain scores (SF-36 scores summarized in Table 2). The combination group fared significantly better in terms of HRQOL improvement than the olanzapine-alone group, showing improvement in 5 of the SF-36 domain scores and in QLDS total score. The authors also performed a psychometric evaluation of the QLDS (see section III).

Finally, the Q-LES-Q has been administered at baseline (hospital discharge), 6 and 12 weeks in a comparison of divalproex sodium and olanzapine in the treatment of acute mania [71]. No significant treatment effects were detected in Q-LES-Q scores in the study, although only 52 (43%) of the 120 patients randomized to either divalproex or olanzapine completed the QoL instrument. Interestingly, the authors reported an association between weight gain being reported as an adverse event and poorer change scores in the physical, leisure, and general activities domains of the Q-LES-Q at 6 weeks (but not at 12 weeks). Negative correlations were reported between increased weight (at 6 weeks) and overall life satisfaction, physical health, mood, general activities and satisfaction with medication on the Q-LES-Q.

Although current recommendations favor the use of pharmacological treatments such as lithium and mood stabilizers in the initial treatment and symptom control of BD, there is increasing recognition of the role of psychotherapy in the management of the disorder. We identified one study that had used a QoL tool to assess outcome following a psychotherapy intervention for BD. Patelis-Siotis and colleagues [72] used the SF-36 in a feasibility study of group cognitive behavior therapy (CBT) in patients with BD. Although baseline SF-36 data was available for 42 patients (see Table 2), pre and post intervention data was only available for a proportion of participants (N = 22) as completion of the QoL questionnaires was optional. Nevertheless, SF-36 vitality and role emotional scores were significantly improved following CBT, with an accompanying trend towards improved social functioning. Another study we identified specifically examined the effects of vocational rehabilitation upon outcome in 149 patients with SMI, 21 of whom were diagnosed with bipolar disorder [73]. In personal communication with the authors, we learned that mean (± SD, range 1–7 where higher scores indicate better QoL) baseline QOLI 'overall life satisfaction' scores were 4.7 ± 1.1, with 'general satisfaction' domain scores of 4.9 ± 1.3. Although outcome data was not available specifically for the bipolar group, better QoL outcomes were associated with 'competitive work activity' in the overall sample compared to other reduced forms of work activity. Finally, a recent study has examined the effects of providing 3 sessions of psychoeducation about lithium treatment to patients (N = 26) with BD [74]. In addition to assessing the effects of psychoeducation upon medication adherence, the authors examined the impact of education upon QoL, as measured by the WHO-QOL-BREF. Following psychoeducation, patients in the intervention arm of the study showed significant improvement in 2 of the WHOQOL BREF's 4 domains (physical health and social functioning) and in overall perceived health. Patients in the control arm of the study, in comparison, showed no significant changes in their perceived QoL. The results of the study indicate that it may be possible to alter patients' perceptions of their QoL even with relatively brief psychological interventions.

## Discussion

Prior to 1999, only 10 studies had systematically addressed the measurement of HRQOL in patients with bipolar disorder [10]. A second review of studies that had examined HRQOL in BD prior to 2004 identified 65 studies [9]. We conducted a subsequent review of studies examining generic and HRQOL in bipolar disorder that had been published prior to November 2004. Our literature search identified 28 studies in total, 7 (25%) of which were published before 1999 (there is discrepancy in the number of studies identified prior to 1999 between the two reviews due to differing inclusion criteria). The remaining 21 (75%) were published between 2000 and 2004, indicating that there is developing interest in this field of research. The studies we identified were quite heterogeneous in nature. Several undertook to assess QoL during different phases of the disorder, for example, cross-sectional research that compared perceived QoL in euthymic, manic or depressed patients with BD. Other studies compared QoL in bipolar samples to that of other patient populations, both with other psychiatric disorders and with chronic physical conditions. Another vein of research examined the psychometric properties of a variety of generic and HRQOL instruments in BD populations. Finally, we identified several studies that had used a QoL instrument to assess outcome in trials of pharmacological and non-pharmacological treatment inventions for the condition.

The studies were also of variable scientific quality. Methodological shortcomings included small sample sizes, cross-sectional designs, idiosyncratic diagnostic methods or undifferentiated diagnostic groups, and use of inappropriate or poorly validated QoL instruments. This being said, the overall scientific quality of research in this field does appear to be improving. Of the 10 studies identified in the review by Namjoshi and colleagues, only one possessed a sample of size of more than 100 patients with BD. In comparison, we identified eleven studies that had enrolled more than 100 patients. It was particularly encouraging to see that some of the large pharmacological trials of treatment interventions for BD are now using QoL measures as secondary outcome measures. Clinical trials in bipolar populations have traditionally used objectively rated measures such as rates of relapse, hospitalization or symptom reduction to assess patient outcome. However, the concomitant use of QoL indices does appear to pay dividends. For example, Namjoshi and colleagues [66] found that the timing of response to treatment with olanzapine differed in terms of symptomatic and QoL outcome. Symptom reduction in the olanzapine group occurred relatively quickly, with patients showing a 10-point decrease in Young Mania Rating Scale (YMRS) scores during the study's 3-week acute treatment phase. Improvements in SF-36 scores, however, occurred more slowly. Only the domain of physical functioning showed significant improvement by the end of the acute treatment phase, whereas it was the domains of social functioning, general health, vitality and bodily pain that were significantly improved at the end of the 49-week maintenance phase. These findings are in accordance with other research showing that 98% of first episode mania patients achieve syndromal recovery after 24 months, but only 38% achieve functional recovery [75]. Sole reliance on symptomatic outcome measures may not detect these more subtle changes in well-being, functioning and QoL.

Although there appears to be increasing use of QoL measures in pharmacological research in bipolar populations, we identified surprisingly few studies of psychological interventions that had incorporated a QoL assessment. In a review of psychosocial interventions for BD, Huxley and colleagues (2000) [76] identified 32 peer-reviewed reports examining group, couple/family or individual psychotherapy in BD, none of which systematically assessed QoL. Our own review only identified one relevant publication, a feasibility study of group CBT which incorporated the SF-36 [72]. As Huxley and colleagues note, future research in this area should employ much broader measures of outcome, such as the assessment of QoL, which may be less amenable to pharmacological treatment in isolation.

An important general conclusion from this review is that the measurement of QoL is feasible in some patients with BD. However, there remains a paucity of information about the ability of patients in the hypo/manic phase of their illness to reliably and accurately complete QoL measures. One of the more rigorous studies to date was performed by Russo and colleagues (1997) [58], in which nurses administered the QOLI via a structured interview procedure to 103 patients with acute mania. Completion rates for the questionnaire were 69% in acutely manic patients, compared to 88% in bipolar depressed patients. In the Namjoshi et al., [66] study of olanzapine in patients with acute mania, SF-36 scores were successfully obtained for 122 of 139 (88%) of patients who entered the study's randomization phase. In the smaller study by Vojta and colleagues [36], two brief QoL instruments were successfully completed by 16 patients with mania/hypomania. More research is needed to ascertain how feasible it is to administer self-report measures of QoL in patients with hypo/mania, although other research in bipolar populations has indicated that patients with mild to moderate manic symptoms can provide reliable descriptions of their symptoms [77]. Although there is controversy about the validity of the technique, [78] proxy measures of QoL can be obtained from family members or clinicians, and may offer one solution to this problem. Additional research is also needed to determine the longitudinal course of QoL in patients with BD. The majority of the studies we identified were cross-sectional in nature. Useful future research would longitudinally follow the course of QoL in a cohort of patients with BD as they experienced different phases of the illness. Research is also required in first-episode mania patients to help elucidate the relationship between length of illness, episode frequency and QoL.

The studies we identified were also heterogeneous in terms of the QoL instruments they incorporated. By far the most frequently utilized were the MOS range of HRQOL measures; 16 of the 28 studies we identified (57%) utilized the SF-12, SF-20 or the SF-36. The results of studies using the SF-36 were amalgamated in Table 2. Inspection of these data indicates that, in general, physical functioning appears to be relatively good in patients with BD (range 63.8 – 91.2). Mental health scores are unsurprisingly much lower (range 30.0 – 72.8). In comparison, SF-36 mental health functioning scores have been reported to be 40.0 (± 17.5) in primary care patients (N = 536) initiating treatment for depression [79]. However, it remains difficult to make any broad generalizations on the basis of this grouped data owing to differences in patient populations, recruitment methods and sample sizes that result in wide ranges of scores in some domains. Given the breadth of existing data for the SF-36 in bipolar populations, the scale's acceptable psychometric properties and detailed normative data, we recommend this scale for the measurement of health-related QoL in patients with BD. The WHO-QOL-BREF is an alternative that has undergone rigorous international development and is available in a wide variety of languages.

A number of other QoL instruments have been utilized in bipolar populations, including the Illness Intrusiveness Rating Scale (IIRS), the Quality of Life Enjoyment and Satisfaction Questionnaire (Q-LES-Q), the Lehman Quality of Life Interview (QOLI), the WHO-QOL-BREF and the health utility time tradeoff (TTO) and standard gamble (SG) approaches. However, only a small number of the studies we identified reported on the psychometric properties of these instruments, and it remains the case that few measures of QoL have been appropriately evaluated for use in BD populations. Some of these instruments are semi disease-targeted in the sense that they have been developed in and for depressed populations (i.e. the Q-LES-Q and QLDS). Both the Q-LES-Q and QLDS appear to possess acceptable psychometric properties and, importantly, are responsive to change in response to both psychological and pharmacological treatment interventions and can be recommended here for use in bipolar populations.

There is at present no QoL measure specifically designed for use in bipolar populations. Although existing QoL instruments are likely to capture key aspects of QoL, they may be insensitive to some of the unique problems posed by this complex psychiatric condition. For example, few QoL instruments designed for use in psychiatric populations assess routine, independence, spirituality or stigma, which have been sown to have particular bearing upon QoL in patients with BD [80]. Bipolar disorder is also unique in that it can be characterized by a variety of mood states, including hypo/mania, depression or mixed states. The understanding that mania can also be associated with depressive symptoms, and that patients will experience periods when they are relatively euthymic, complicates the assessment of QoL in this population. We suggest that an important step forward in this field of research would be made with the development of a disease-specific QoL instrument for BD. We believe that such an instrument would need to have a number of qualities. It would have to work effectively in the depressed, hypo/manic, mixed and euthymic phases of BD. It would need to be concise enough not to put overdue response burden on the patient, but detailed enough to tap into the major areas of well-being affected by the disorder. The relevance of the scale would need to be ensured by thorough consultation with patients, their families and their clinicians. This process should involve individual qualitative interviews and focus group work with patients with BD and their family members at different stages of the disorder, and consultation with psychiatrists, mental health workers, and public-sector organizations. It would be useful if the instrument was available in self-report, interviewer-administered and proxy-respondent formats to provide alternative methods of administration in acutely manic populations. Finally, the psychometric properties of the instrument would need to be carefully evaluated in terms of reliability, validity, responsiveness and other standard psychometric assessments.

## Conclusion

In recent years, major developments in the pharmacological control of bipolar disorder have occurred. One result of these improvements has been that some patients will BD now experience fewer side effects and less physical symptomatology, allowing the focus to shift to other concerns, including improving inter-episode functioning and perceived quality of life. Our review found that there is growing interest in characterizing QoL in bipolar disorder populations, and determining the impact of treatment interventions upon life quality. The scientific quality of research in this field has been variable, but increasing numbers of studies of good design are now being conducted. We highlighted several important avenues for future research, including the need for assessments of QoL in first episode and hypo/manic patients, more well-designed longitudinal research and research into the impact of psychosocial interventions upon QoL, and the development of a disease-specific measure for use in bipolar populations. Bipolar disorder creates a major health concern, both for the individual and for society, and more information is still needed about the impact of the condition upon QoL. Such information may then bring us one step closer towards developing treatment regimens that maximize both symptom reduction and quality of life for patients with this complex condition.
